# Crystal structure of 5-di­ethyl­amino-2-({[4-(di­ethyl­amino)­phen­yl]imino}­meth­yl)phenol

**DOI:** 10.1107/S205698901501645X

**Published:** 2015-09-12

**Authors:** C. Vidya Rani, G. Chakkaravarthi, N. Indra Gandhi, G. Rajagopal

**Affiliations:** a PG & Research Department of Chemistry, Chikkanna Government Arts College, Tiruppur 641 602, India; b Department of Physics, CPCL Polytechnic College, Chennai 600 068, India; c PG & Research Department of Chemistry, Presidency College (Autonomous), Chennai 600 005, India

**Keywords:** crystal structure, phenol, Schiff base, intra­molecular hydrogen bond, C—H⋯π inter­actions, biological activity, pharmacological activity

## Abstract

In the title compound, C_21_H_29_N_3_O, the dihedral angle between the planes of the aromatic rings is 8.1 (2)°. The ethyl groups at one terminal site of the compound are disordered over two sets of sites with occupancies of 0.775 (9) and 0.225 (9). The mol­ecule has an *E* conformation about the N=C bond. The mol­ecular structure features an intra­molecular O—H⋯N hydrogen bond, which closes an *S*(6) loop. In the crystal, weak C—H⋯π inter­actions leads to the formation of a three-dimensional network.

## Related literature   

For biological and pharmacological activities of Schiff base compounds and their derivatives, see: Khandar *et al.* (2005 ▸); Chen *et al.* (2006 ▸); Kidwai *et al.* (2000 ▸). For similar structures, see: Manvizhi *et al.* (2011 ▸); Thirugnanasundar *et al.* (2011 ▸); Rani *et al.* (2015 ▸).
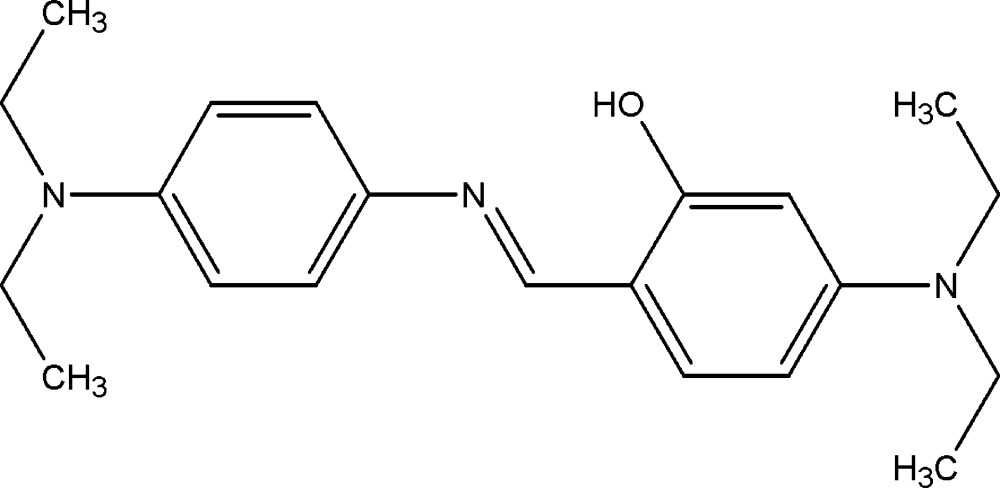


## Experimental   

### Crystal data  

C_21_H_29_N_3_O
*M* *_r_* = 339.47
Orthorhombic, 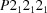

*a* = 8.1986 (4) Å
*b* = 9.7128 (4) Å
*c* = 24.4172 (12) Å
*V* = 1944.38 (16) Å^3^
*Z* = 4
Mo *K*α radiation
μ = 0.07 mm^−1^
*T* = 295 K
0.28 × 0.26 × 0.24 mm

### Data collection  

Bruker Kappa APEX II CCD diffractometer
Absorption correction: multi-scan (*SADABS*; Sheldrick, 1996 ▸) *T*_min_ = 0.980, *T*_max_ = 0.983
29557 measured reflections
3556 independent reflections
2130 reflections with *I* > 2σ(*I*)
*R*_int_ = 0.046

### Refinement  

*R*[*F* ^2^ > 2σ(*F* ^2^)] = 0.064
*wR*(*F* ^2^) = 0.205
*S* = 1.07
3556 reflections
272 parameters
10 restraints
H atoms treated by a mixture of independent and constrained refinement
Δρ_max_ = 0.44 e Å^−3^
Δρ_min_ = −0.20 e Å^−3^

### 

Data collection: *APEX2* (Bruker, 2004 ▸); cell refinement: *SAINT* (Bruker, 2004 ▸); data reduction: *SAINT*; program(s) used to solve structure: *SHELXS97* (Sheldrick, 2008 ▸); program(s) used to refine structure: *SHELXL97* (Sheldrick, 2008 ▸); molecular graphics: *PLATON* (Spek, 2009 ▸); software used to prepare material for publication: *SHELXL97* and *PLATON* (Spek, 2009 ▸).

## Supplementary Material

Crystal structure: contains datablock(s) global, I. DOI: 10.1107/S205698901501645X/rk2432sup1.cif

Structure factors: contains datablock(s) I. DOI: 10.1107/S205698901501645X/rk2432Isup2.hkl

Supporting information file. DOI: 10.1107/S205698901501645X/rk2432Isup3.cml

**Fig. 1.**
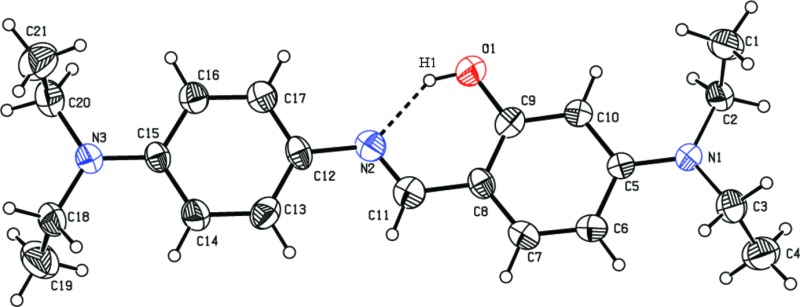
The molecular structure of title compaund, with the atom–numbering scheme. Displacement ellipsoids are drawn at the 30% probability level. H atoms are presented as a small spheres of arbitrary radius. The intramolecular hydrogen bond is depicted by a dashed line. Only the major occupancy component of the disordered diethylamino–group [—N1(C2H5)2] is shown.

2 5 2 . DOI: 10.1107/S205698901501645X/rk2432fig1.tif
The mol­ecular structure of title compaund, with the atom–numbering scheme. Displacement ellipsoids are drawn at the 30% probability level. H atoms are presented as a small spheres of arbitrary radius. The intra­molecular hydrogen bond is depicted by a dashed line. Only the major occupancy component of the disordered di­ethyl­amino–group [—N1(C_2_H_5_)_2_] is shown.

CCDC reference: 1422036

Additional supporting information:  crystallographic information; 3D view; checkCIF report

## Figures and Tables

**Table 1:** Hydrogen-bond geometry (, ) *Cg*1 and *Cg*2 are the centroids of the C5C10 and C12C17 rings, respectively.

*D*H*A*	*D*H	H*A*	*D* *A*	*D*H*A*
O1H1N2	0.86(2)	1.81(4)	2.563(5)	144(6)
C18H18*A* *Cg*2^i^	0.97	2.92	3.660(5)	134
C1*A*H1*A*1*Cg*1^ii^	0.96	2.80	3.49(4)	130

Symmetry codes: (i) 
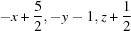
; (ii) 
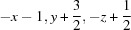
.

## supplementary crystallographic information

### S1. Comment

Schiff base derivatives serve as intermediates in certain enzymatic reactions and are also found in proteins that form the connective tissue (Khandar *et al.*, 2005; Chen *et al.*, 2006) and in the pharmaceutical field (Kidwai *et al.*, 2000). We herein report the crystal structure of the title compound (Fig.1). The geometric parameters of the title compound are comparable to the reported structures (Manvizhi *et al.*, 2011; Thirugnanasundar *et al.*, 2011; Rani *et al.*, 2015). The dihedral angle between the rings (C5–C10) and (C12–C17) is 8.1 (2)°. The ethyl groups at one terminal site (N1) of the compound are disordered over two positions, with the site occupancies of 0.775 (9) and 0.225 (9). The molecular structure is stabilized by weak intramolecular O—H···N hydrogen bond (Table 1). The crystal structure is influenced by weak C—H···π (Table 1) interactions to form a three dimensional network.

### S2. Experimental

For the preparation of Schiff base, an ethanolic solution (10 ml) of 5–(diethylamino)–2–hydroxybenzaldehyde (0.5 mol) and the same volume of ethanolic solution of *N*,*N*–diethylbenzene–1,4–diamine (0.5 mol) are mixed. The solution is mixed on magnetic stirrer with addition of 2 to 3 drops of glacial acetic acid. The reaction mixture is refluxed for 2 hrs and allowed to cool down to room temperature, crystalline solid precipitate from the mixture is separated out. Crystalline products are washed with ice cold ethanol and dried *in vacuo* over anhydrous CaCl_2_. Single crystals suitable for the X-ray diffraction are obtained by slow evaporation of a solution of the title compound in *DMF* at room temperature.

### S3. Refinement

The H atoms were positioned geometrically and refined using riding model with C—H = 0.93 Å and *U*_iso_(H) = 1.2*U*_eq_(C) for aromatic H, C—H = 0.97 Å and *U*_iso_(H) = 1.2*U*_eq_(C) for CH_2_, C—H = 0.96 Å and *U*_iso_(H) = 1.5*U*_eq_(C) for CH_3_. H atom for O atom is found from Fourier map and refined freely with *U*_iso_(H) = 1.5*U*_eq_(O) and distance restraint 0.82 Å. The components of the anisotropic displacement parameters in the direction of the bond between C9 and O1 were restrained to be equal within an effective standard deviation of 0.001 using the DELU command. The N1—C2, N1—C3, N1—C2A, N1—C3A distances were restraint to 1.46 (1) Å and C1—C2, C1A—C2A, C3—C4 and C3A—C4A distances were restraint to 1.53 (1) Å

### Crystal data

**Table:**

C_21_H_29_N_3_O	*F*(000) = 736
*M**_r_* = 339.47	*D*_x_ = 1.160 Mg m^−^^3^
Orthorhombic, *P*2_1_2_1_2_1_	Mo *K*α radiation, λ = 0.71073 Å
Hall symbol: P 2ac 2ab	Cell parameters from 7089 reflections
*a* = 8.1986 (4) Å	θ = 2.5–25.3°
*b* = 9.7128 (4) Å	µ = 0.07 mm^−^^1^
*c* = 24.4172 (12) Å	*T* = 295 K
*V* = 1944.38 (16) Å^3^	Block, colourless
*Z* = 4	0.28 × 0.26 × 0.24 mm

### Data collection

**Table:**

Bruker Kappa APEX II CCD diffractometer	3556 independent reflections
Radiation source: fine–focus sealed tube	2130 reflections with *I* > 2σ(*I*)
Graphite monochromator	*R*_int_ = 0.046
ω and φ scans	θ_max_ = 25.4°, θ_min_ = 2.3°
Absorption correction: multi-scan (*SADABS*; Sheldrick, 1996)	*h* = −9→9
*T*_min_ = 0.980, *T*_max_ = 0.983	*k* = −11→11
29557 measured reflections	*l* = −29→29

### Refinement

**Table:**

Refinement on *F*^2^	Secondary atom site location: difference Fourier map
Least-squares matrix: full	Hydrogen site location: inferred from neighbouring sites
*R*[*F*^2^ > 2σ(*F*^2^)] = 0.064	H atoms treated by a mixture of independent and constrained refinement
*wR*(*F*^2^) = 0.205	*w* = 1/[σ^2^(*F*_o_^2^) + (0.0727*P*)^2^ + 1.5032*P*] where *P* = (*F*_o_^2^ + 2*F*_c_^2^)/3
*S* = 1.07	(Δ/σ)_max_ < 0.001
3556 reflections	Δρ_max_ = 0.44 e Å^−^^3^
272 parameters	Δρ_min_ = −0.20 e Å^−^^3^
10 restraints	Absolute structure: Flack (1983), 1466 Friedel pairs
Primary atom site location: structure-invariant direct methods	

### Special details

**Table:**

Geometry. All s.u.'s (except the s.u. in the dihedral angle between two l.s. planes) are estimated using the full covariance matrix. The cell s.u.'s are taken into account individually in the estimation of s.u.'s in distances, angles and torsion angles; correlations between s.u.'s in cell parameters are only used when they are defined by crystal symmetry. An approximate (isotropic) treatment of cell s.u.'s is used for estimating s.u.'s involving l.s. planes.
Refinement. Refinement of *F*^2^ against ALL reflections. The weighted *R*–factor *wR* and goodness of fit *S* are based on *F*^2^, conventional *R*–factors *R* are based on *F*, with *F* set to zero for negative *F*^2^. The threshold expression of *F*^2^ > σ(*F*^2^) is used only for calculating *R*–factors(gt) *etc*. and is not relevant to the choice of reflections for refinement. *R*–factors based on *F*^2^ are statistically about twice as large as those based on *F*, and *R*–factors based on ALL data will be even larger.

### Fractional atomic coordinates and isotropic or equivalent isotropic displacement parameters (Å^2^)

**Table:**

	*x*	*y*	*z*	*U*_iso_*/*U*_eq_	Occ. (<1)
C1	0.7266 (10)	0.9101 (7)	−0.0244 (3)	0.084 (2)	0.775 (9)
H1A	0.7411	0.8690	0.0111	0.125*	0.775 (9)
H1B	0.6538	0.9871	−0.0214	0.125*	0.775 (9)
H1C	0.8302	0.9409	−0.0380	0.125*	0.775 (9)
C2	0.6555 (11)	0.8049 (8)	−0.0633 (4)	0.067 (3)	0.775 (9)
H2A	0.5577	0.7651	−0.0475	0.080*	0.775 (9)
H2B	0.6260	0.8492	−0.0975	0.080*	0.775 (9)
C3	0.9082 (10)	0.7205 (8)	−0.1156 (3)	0.073 (2)	0.775 (9)
H3A	0.9375	0.8172	−0.1161	0.088*	0.775 (9)
H3B	1.0047	0.6674	−0.1065	0.088*	0.775 (9)
C4	0.8438 (10)	0.6778 (8)	−0.1703 (3)	0.093 (3)	0.775 (9)
H4A	0.8211	0.5809	−0.1699	0.140*	0.775 (9)
H4B	0.9237	0.6975	−0.1979	0.140*	0.775 (9)
H4C	0.7454	0.7277	−0.1780	0.140*	0.775 (9)
C1A	0.586 (4)	0.847 (5)	−0.0615 (19)	0.122 (16)	0.225 (9)
H1A1	0.5521	0.9397	−0.0545	0.183*	0.225 (9)
H1A2	0.5193	0.7847	−0.0405	0.183*	0.225 (9)
H1A3	0.5730	0.8267	−0.0998	0.183*	0.225 (9)
C2A	0.764 (4)	0.8291 (15)	−0.0453 (9)	0.085 (9)	0.225 (9)
H2A1	0.8345	0.8993	−0.0607	0.102*	0.225 (9)
H2A2	0.7798	0.8221	−0.0061	0.102*	0.225 (9)
C3A	0.787 (3)	0.6858 (16)	−0.1341 (4)	0.061 (7)	0.225 (9)
H3A1	0.7019	0.7390	−0.1519	0.073*	0.225 (9)
H3A2	0.7824	0.5911	−0.1467	0.073*	0.225 (9)
C4A	0.955 (3)	0.749 (3)	−0.1425 (13)	0.080 (9)	0.225 (9)
H4A1	0.9478	0.8474	−0.1389	0.120*	0.225 (9)
H4A2	0.9944	0.7263	−0.1783	0.120*	0.225 (9)
H4A3	1.0288	0.7136	−0.1154	0.120*	0.225 (9)
C5	0.7811 (6)	0.5793 (4)	−0.04177 (17)	0.0666 (12)	
C6	0.8764 (6)	0.4644 (5)	−0.05697 (19)	0.0725 (13)	
H6	0.9326	0.4651	−0.0901	0.087*	
C7	0.8865 (6)	0.3522 (5)	−0.02341 (19)	0.0680 (12)	
H7	0.9509	0.2782	−0.0342	0.082*	
C8	0.8054 (5)	0.3444 (4)	0.02559 (16)	0.0541 (10)	
C9	0.7068 (6)	0.4550 (5)	0.03971 (16)	0.0613 (11)	
C10	0.6941 (6)	0.5712 (4)	0.00699 (17)	0.0662 (12)	
H10	0.6274	0.6438	0.0177	0.079*	
C11	0.8246 (6)	0.2245 (5)	0.06113 (19)	0.0643 (12)	
H11	0.8909	0.1522	0.0499	0.077*	
C12	0.7727 (5)	0.1017 (4)	0.14256 (17)	0.0574 (11)	
C13	0.8522 (6)	−0.0207 (5)	0.12960 (18)	0.0706 (13)	
H13	0.9012	−0.0306	0.0955	0.085*	
C14	0.8591 (6)	−0.1287 (5)	0.16721 (18)	0.0679 (12)	
H14	0.9130	−0.2094	0.1577	0.082*	
C15	0.7872 (5)	−0.1183 (4)	0.21878 (16)	0.0515 (10)	
C16	0.7109 (5)	0.0078 (4)	0.23007 (17)	0.0616 (11)	
H16	0.6638	0.0210	0.2643	0.074*	
C17	0.7033 (5)	0.1122 (5)	0.19253 (18)	0.0622 (11)	
H17	0.6487	0.1929	0.2017	0.075*	
C18	0.8789 (6)	−0.3514 (5)	0.2453 (2)	0.0726 (13)	
H18A	0.9128	−0.3912	0.2799	0.087*	
H18B	0.9765	−0.3306	0.2244	0.087*	
C19	0.7787 (9)	−0.4567 (6)	0.2141 (2)	0.109 (2)	
H19A	0.6855	−0.4825	0.2356	0.164*	
H19B	0.8443	−0.5366	0.2070	0.164*	
H19C	0.7429	−0.4176	0.1801	0.164*	
C20	0.7125 (6)	−0.2143 (5)	0.3091 (2)	0.0764 (14)	
H20A	0.6822	−0.3058	0.3213	0.092*	
H20B	0.6132	−0.1607	0.3054	0.092*	
C21	0.8191 (9)	−0.1487 (7)	0.3515 (2)	0.113 (2)	
H21A	0.9208	−0.1975	0.3535	0.170*	
H21B	0.7657	−0.1521	0.3864	0.170*	
H21C	0.8393	−0.0546	0.3417	0.170*	
N1	0.7767 (7)	0.6954 (4)	−0.07378 (16)	0.1047 (19)	
N2	0.7542 (4)	0.2174 (4)	0.10594 (14)	0.0626 (10)	
N3	0.7918 (5)	−0.2243 (4)	0.25593 (14)	0.0654 (10)	
O1	0.6208 (5)	0.4508 (4)	0.08624 (14)	0.0858 (11)	
H1	0.643 (8)	0.379 (4)	0.105 (2)	0.129*	

### Atomic displacement parameters (Å^2^)

**Table:**

	*U*^11^	*U*^22^	*U*^33^	*U*^12^	*U*^13^	*U*^23^
C1	0.091 (5)	0.071 (5)	0.089 (5)	0.010 (4)	0.006 (4)	−0.013 (4)
C2	0.084 (8)	0.057 (5)	0.059 (4)	0.014 (5)	−0.005 (5)	0.004 (3)
C3	0.081 (6)	0.061 (4)	0.078 (6)	−0.007 (4)	0.005 (4)	0.004 (4)
C4	0.099 (6)	0.105 (6)	0.075 (5)	0.017 (5)	0.000 (4)	−0.008 (4)
C1A	0.11 (3)	0.11 (3)	0.15 (3)	−0.01 (2)	0.04 (3)	0.01 (3)
C2A	0.12 (3)	0.08 (2)	0.058 (16)	−0.004 (18)	−0.006 (17)	0.015 (14)
C3A	0.13 (2)	0.021 (8)	0.036 (11)	0.008 (11)	0.021 (12)	0.000 (7)
C4A	0.076 (17)	0.080 (19)	0.09 (2)	−0.020 (14)	0.023 (15)	−0.010 (16)
C5	0.091 (3)	0.054 (3)	0.055 (2)	0.012 (3)	0.019 (3)	0.003 (2)
C6	0.090 (4)	0.061 (3)	0.067 (3)	0.008 (3)	0.018 (3)	−0.006 (2)
C7	0.075 (3)	0.054 (3)	0.075 (3)	0.010 (2)	0.004 (3)	−0.005 (2)
C8	0.050 (2)	0.048 (2)	0.064 (3)	0.000 (2)	−0.010 (2)	0.0036 (19)
C9	0.059 (2)	0.071 (3)	0.054 (2)	−0.004 (2)	0.0005 (17)	0.001 (2)
C10	0.081 (3)	0.059 (3)	0.059 (2)	0.017 (3)	0.015 (2)	0.005 (2)
C11	0.056 (3)	0.071 (3)	0.065 (3)	−0.002 (2)	−0.012 (2)	−0.003 (2)
C12	0.052 (2)	0.049 (2)	0.071 (3)	0.000 (2)	−0.017 (2)	0.010 (2)
C13	0.081 (3)	0.080 (3)	0.050 (2)	−0.005 (3)	−0.002 (2)	0.002 (2)
C14	0.078 (3)	0.061 (3)	0.065 (3)	0.013 (2)	−0.007 (2)	0.006 (2)
C15	0.051 (2)	0.046 (2)	0.058 (2)	−0.002 (2)	−0.011 (2)	0.0053 (19)
C16	0.056 (2)	0.062 (3)	0.067 (3)	0.001 (2)	−0.003 (2)	0.004 (2)
C17	0.056 (3)	0.064 (3)	0.067 (3)	0.001 (2)	−0.006 (2)	0.002 (2)
C18	0.077 (3)	0.063 (3)	0.077 (3)	0.015 (3)	−0.004 (3)	0.014 (3)
C19	0.144 (6)	0.066 (3)	0.116 (4)	0.001 (4)	−0.014 (5)	−0.014 (3)
C20	0.073 (3)	0.063 (3)	0.093 (3)	−0.001 (3)	0.001 (3)	0.018 (3)
C21	0.145 (6)	0.123 (5)	0.072 (3)	−0.005 (5)	−0.001 (4)	−0.008 (3)
N1	0.176 (5)	0.065 (3)	0.072 (3)	0.038 (3)	0.061 (3)	0.020 (2)
N2	0.061 (2)	0.065 (2)	0.061 (2)	0.0032 (19)	−0.0139 (19)	−0.0030 (18)
N3	0.081 (2)	0.054 (2)	0.062 (2)	0.011 (2)	0.000 (2)	0.0053 (17)
O1	0.105 (3)	0.084 (2)	0.068 (2)	0.028 (2)	0.0249 (18)	0.0131 (18)

### Geometric parameters (Å, º)

**Table:**

C1—C2	1.513 (8)	C8—C9	1.388 (6)
C1—H1A	0.9600	C8—C11	1.461 (6)
C1—H1B	0.9600	C9—O1	1.338 (5)
C1—H1C	0.9600	C9—C10	1.387 (6)
C2—N1	1.478 (7)	C10—H10	0.9300
C2—H2A	0.9700	C11—N2	1.239 (5)
C2—H2B	0.9700	C11—H11	0.9300
C3—C4	1.495 (7)	C12—C17	1.350 (6)
C3—N1	1.504 (7)	C12—C13	1.392 (6)
C3—H3A	0.9700	C12—N2	1.444 (5)
C3—H3B	0.9700	C13—C14	1.395 (6)
C4—H4A	0.9600	C13—H13	0.9300
C4—H4B	0.9600	C14—C15	1.394 (6)
C4—H4C	0.9600	C14—H14	0.9300
C1A—C2A	1.523 (10)	C15—N3	1.373 (5)
C1A—H1A1	0.9600	C15—C16	1.403 (6)
C1A—H1A2	0.9600	C16—C17	1.368 (6)
C1A—H1A3	0.9600	C16—H16	0.9300
C2A—N1	1.477 (10)	C17—H17	0.9300
C2A—H2A1	0.9700	C18—N3	1.450 (5)
C2A—H2A2	0.9700	C18—C19	1.516 (7)
C3A—N1	1.478 (9)	C18—H18A	0.9700
C3A—C4A	1.524 (10)	C18—H18B	0.9700
C3A—H3A1	0.9700	C19—H19A	0.9600
C3A—H3A2	0.9700	C19—H19B	0.9600
C4A—H4A1	0.9600	C19—H19C	0.9600
C4A—H4A2	0.9600	C20—N3	1.455 (6)
C4A—H4A3	0.9600	C20—C21	1.497 (7)
C5—N1	1.372 (5)	C20—H20A	0.9700
C5—C10	1.390 (6)	C20—H20B	0.9700
C5—C6	1.412 (6)	C21—H21A	0.9600
C6—C7	1.366 (6)	C21—H21B	0.9600
C6—H6	0.9300	C21—H21C	0.9600
C7—C8	1.371 (6)	O1—H1	0.86 (2)
C7—H7	0.9300		
			
N1—C2—C1	109.6 (7)	C17—C12—C13	117.8 (4)
N1—C2—H2A	109.8	C17—C12—N2	117.2 (4)
C1—C2—H2A	109.8	C13—C12—N2	125.0 (4)
N1—C2—H2B	109.8	C12—C13—C14	120.8 (4)
C1—C2—H2B	109.8	C12—C13—H13	119.6
H2A—C2—H2B	108.2	C14—C13—H13	119.6
C4—C3—N1	107.9 (7)	C15—C14—C13	121.5 (4)
C4—C3—H3A	110.1	C15—C14—H14	119.2
N1—C3—H3A	110.1	C13—C14—H14	119.2
C4—C3—H3B	110.1	N3—C15—C14	122.1 (4)
N1—C3—H3B	110.1	N3—C15—C16	122.5 (4)
H3A—C3—H3B	108.4	C14—C15—C16	115.4 (4)
C2A—C1A—H1A1	109.5	C17—C16—C15	122.4 (4)
C2A—C1A—H1A2	109.5	C17—C16—H16	118.8
H1A1—C1A—H1A2	109.5	C15—C16—H16	118.8
C2A—C1A—H1A3	109.5	C12—C17—C16	122.0 (4)
H1A1—C1A—H1A3	109.5	C12—C17—H17	119.0
H1A2—C1A—H1A3	109.5	C16—C17—H17	119.0
N1—C2A—C1A	93 (2)	N3—C18—C19	113.4 (4)
N1—C2A—H2A1	113.2	N3—C18—H18A	108.9
C1A—C2A—H2A1	113.2	C19—C18—H18A	108.9
N1—C2A—H2A2	113.2	N3—C18—H18B	108.9
C1A—C2A—H2A2	113.2	C19—C18—H18B	108.9
H2A1—C2A—H2A2	110.5	H18A—C18—H18B	107.7
N1—C3A—C4A	99.1 (15)	C18—C19—H19A	109.5
N1—C3A—H3A1	111.9	C18—C19—H19B	109.5
C4A—C3A—H3A1	111.9	H19A—C19—H19B	109.5
N1—C3A—H3A2	111.9	C18—C19—H19C	109.5
C4A—C3A—H3A2	111.9	H19A—C19—H19C	109.5
H3A1—C3A—H3A2	109.6	H19B—C19—H19C	109.5
C3A—C4A—H4A1	109.5	N3—C20—C21	112.6 (4)
C3A—C4A—H4A2	109.5	N3—C20—H20A	109.1
H4A1—C4A—H4A2	109.5	C21—C20—H20A	109.1
C3A—C4A—H4A3	109.5	N3—C20—H20B	109.1
H4A1—C4A—H4A3	109.5	C21—C20—H20B	109.1
H4A2—C4A—H4A3	109.5	H20A—C20—H20B	107.8
N1—C5—C10	121.4 (4)	C20—C21—H21A	109.5
N1—C5—C6	120.9 (4)	C20—C21—H21B	109.5
C10—C5—C6	117.7 (4)	H21A—C21—H21B	109.5
C7—C6—C5	120.4 (4)	C20—C21—H21C	109.5
C7—C6—H6	119.8	H21A—C21—H21C	109.5
C5—C6—H6	119.8	H21B—C21—H21C	109.5
C6—C7—C8	122.6 (4)	C5—N1—C2A	117.2 (10)
C6—C7—H7	118.7	C5—N1—C3A	120.9 (7)
C8—C7—H7	118.7	C2A—N1—C3A	121.9 (12)
C7—C8—C9	117.2 (4)	C5—N1—C2	120.7 (5)
C7—C8—C11	120.7 (4)	C2A—N1—C2	40.3 (12)
C9—C8—C11	122.1 (4)	C3A—N1—C2	104.8 (9)
O1—C9—C10	118.3 (4)	C5—N1—C3	120.0 (5)
O1—C9—C8	119.7 (4)	C2A—N1—C3	103.2 (12)
C10—C9—C8	122.0 (4)	C3A—N1—C3	45.1 (8)
C9—C10—C5	120.1 (4)	C2—N1—C3	118.8 (6)
C9—C10—H10	120.0	C11—N2—C12	122.8 (4)
C5—C10—H10	120.0	C15—N3—C18	122.3 (4)
N2—C11—C8	121.3 (4)	C15—N3—C20	121.8 (4)
N2—C11—H11	119.4	C18—N3—C20	115.9 (4)
C8—C11—H11	119.4	C9—O1—H1	112 (4)
			
N1—C5—C6—C7	176.6 (5)	C6—C5—N1—C2	169.0 (6)
C10—C5—C6—C7	−2.5 (8)	C10—C5—N1—C3	160.6 (5)
C5—C6—C7—C8	0.6 (8)	C6—C5—N1—C3	−18.5 (8)
C6—C7—C8—C9	1.8 (7)	C1A—C2A—N1—C5	−106 (2)
C6—C7—C8—C11	−177.7 (4)	C1A—C2A—N1—C3A	75 (3)
C7—C8—C9—O1	177.9 (4)	C1A—C2A—N1—C2	0 (2)
C11—C8—C9—O1	−2.7 (6)	C1A—C2A—N1—C3	120 (2)
C7—C8—C9—C10	−2.2 (6)	C4A—C3A—N1—C5	−110.9 (14)
C11—C8—C9—C10	177.2 (4)	C4A—C3A—N1—C2A	68 (2)
O1—C9—C10—C5	−179.8 (4)	C4A—C3A—N1—C2	108.3 (15)
C8—C9—C10—C5	0.3 (7)	C4A—C3A—N1—C3	−7.8 (14)
N1—C5—C10—C9	−177.1 (5)	C1—C2—N1—C5	91.1 (9)
C6—C5—C10—C9	2.1 (7)	C1—C2—N1—C2A	−5.8 (16)
C7—C8—C11—N2	178.7 (4)	C1—C2—N1—C3A	−128.1 (9)
C9—C8—C11—N2	−0.8 (6)	C1—C2—N1—C3	−81.6 (8)
C17—C12—C13—C14	0.2 (6)	C4—C3—N1—C5	99.5 (7)
N2—C12—C13—C14	−177.9 (4)	C4—C3—N1—C2A	−127.9 (12)
C12—C13—C14—C15	0.0 (7)	C4—C3—N1—C3A	−5.7 (10)
C13—C14—C15—N3	179.3 (4)	C4—C3—N1—C2	−87.8 (7)
C13—C14—C15—C16	−1.0 (6)	C8—C11—N2—C12	−178.8 (4)
N3—C15—C16—C17	−178.5 (4)	C17—C12—N2—C11	173.3 (4)
C14—C15—C16—C17	1.8 (6)	C13—C12—N2—C11	−8.6 (6)
C13—C12—C17—C16	0.7 (6)	C14—C15—N3—C18	3.3 (7)
N2—C12—C17—C16	178.9 (4)	C16—C15—N3—C18	−176.3 (4)
C15—C16—C17—C12	−1.7 (7)	C14—C15—N3—C20	−178.2 (4)
C10—C5—N1—C2A	34.3 (15)	C16—C15—N3—C20	2.1 (6)
C6—C5—N1—C2A	−144.8 (15)	C19—C18—N3—C15	−86.1 (5)
C10—C5—N1—C3A	−146.5 (10)	C19—C18—N3—C20	95.3 (5)
C6—C5—N1—C3A	34.4 (12)	C21—C20—N3—C15	−86.4 (6)
C10—C5—N1—C2	−11.9 (9)	C21—C20—N3—C18	92.1 (5)

### Hydrogen-bond geometry (Å, º)

Cg1 and Cg2 are the centroids of the C5–C10 and C12–C17 rings, respectively.

**Table:**

*D*—H···*A*	*D*—H	H···*A*	*D*···*A*	*D*—H···*A*
O1—H1···N2	0.86 (2)	1.81 (4)	2.563 (5)	144 (6)
C18—H18*A*···*Cg*2^i^	0.97	2.92	3.660 (5)	134
C1*A*—H1*A*1···*Cg*1^ii^	0.96	2.80	3.49 (4)	130

Symmetry codes: (i) −*x*+5/2, −*y*−1, *z*+1/2; (ii) −*x*−1, *y*+3/2, −*z*+1/2.
