# Supplementary figures and images for: Crystal structure of 5-di­ethyl­amino-2-({[4-(di­ethyl­amino)­phen­yl]imino}­meth­yl)phenol
Source: Acta Crystallogr E Crystallogr Commun. 2015 Sep 12;71(Pt 10):o712–3. doi: 10.1107/S205698901501645X (PMC4647405; doi:10.1107/S205698901501645X)

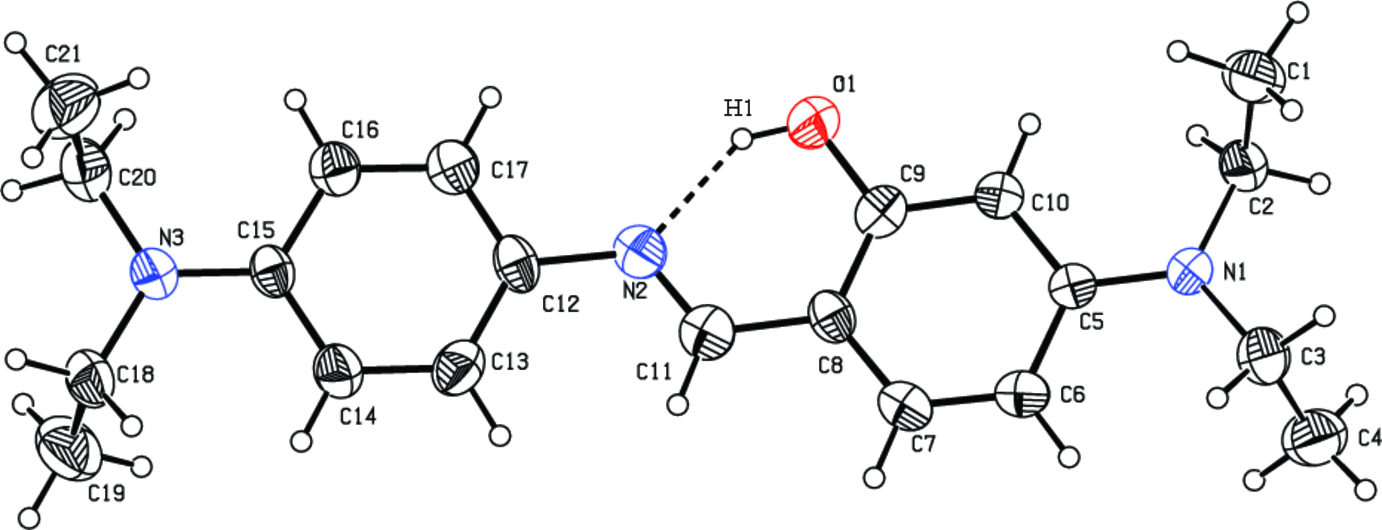

Supplement: Supplementary file 4 [file e-71-0o712-fig1.tif]
